# Why do students skip classroom lectures: A single dental school report

**DOI:** 10.1186/s12909-021-02824-3

**Published:** 2021-07-20

**Authors:** Waleed A. Alamoudi, Azza F. Alhelo, Soulafa A. Almazrooa, Osama M. Felemban, Nada O. Binmadi, Nada A. Alhindi, Sarah A. Ali, Sara K. Akeel, Sana A. Alhamed, Ghadah M. Mansour, Hani H. Mawardi

**Affiliations:** 1 Department of Oral Diagnostic Sciences, King Abdul-Aziz University- Faculty of Dentistry, Jeddah, Saudi Arabia; 2 Department of Pediatric Dentistry, King Abdulaziz University- Faculty of Dentistry, Jeddah, Saudi Arabia

**Keywords:** Classroom, Attendance, Lecture, Absence, Dental students, And dental school

## Abstract

**Background:**

Conventional classroom lectures continue to represent a major component of the dental education system to ensure optimum delivery of knowledge. Certain number of students are less compliant and likely to skip classes which may impact the overall academic performance. The aim of this study was to investigate dental students’ attitude towards classroom attendance and potential reasons for absenteeism at King Abdulaziz University-Faculty of Dentistry (KAU-FD).

**Methods:**

This was a cross-sectional survey of all dental students actively enrolled at KAU-FD from January to June 2019. The survey included questions on demographics, average travel time to school, current dental year, most recent GPA, student’s perspective toward classroom lectures. The survey was validated and distributed to all students at a pre-selected time frame. Data were analysed and presented as frequencies and percentages; chi-square test was used to explore parameters association.

**Results:**

A total of 678 students consented and completed the survey. Overall, 44.3% of students were more likely to skip two classes or less per month. Second year dental students were more likely to be absent from classroom lectures (31.3%), while 3rd year dental students were less likely to do so (15.4%). Reported students’ justifications for missing classes included early morning classes (47.9%), exams preparation (42%), and lecturer’s weak presentation skills (41.9%).

**Conclusion:**

Compliance of dental students with classroom attendance has been an ongoing challenge for most programs. The current data suggests a multifactorial module for students’ attitude toward classroom attendance. Future studies focusing on reasons behind classroom attendance behavior and addressing students’ concerns are needed.

**Supplementary Information:**

The online version contains supplementary material available at 10.1186/s12909-021-02824-3.

## Background

Education, as other aspects of human revolution, has undergone major advances to improve students’ experiences and outcomes [1]. Dental education is one, which followed the same theme and incorporated several innovative learning portals such as clinical hands-on sessions, field trips, critical thinking as well as group discussions [1]. Yet, conventional classroom lectures continue to represent a major component of the educational system to ensure optimum delivery of dental knowledge. In addition, evaluating student performance by examinations and subsequently through course grading continue to be the most common method used to assess effectiveness of teaching and learning [2]. The current literature has suggested numerous variables to affect student performance including social and psychological climate, in addition to academic related factors including intellectual ability, study patterns, instructional methods and learning resources [2, 3].

Over the years, students’ compliance with classroom attendance and engagement in classroom discussions have played a fundamental role in the educational process [1]. However, students’ attendance in classrooms had declined significantly in the recent years on a local and national levels [4]. This maybe attributed in part to change in attitude, their ability to learn the material on their own and today’s era of evolving online education rendering class attendance unneeded [5]. Other factors linked to students’ interest in attending classroom lectures include class subject (the scope and difficulty of the subject), faculty member lecturing style and transportation to school, stress, time management and poor health were also reported as secondary factors contributing to classroom attendance [4, 6, 7]. As a result, a growing controversy over the effectiveness of traditional face-to-face lectures and their contribution to students’ learning is on-going [5].

The frequency of classroom absence may vary across schools, colleges and universities based on geographical and cultural factors [3]. Reviewing the available literature, missing classroom lectures was not uncommon and reported in up to 75% of medical students [7]. At King Abdulaziz University-Faculty of Dentistry (KAU-FD), the dental program is a six-year program which is accredited by Commission on Dental Accreditation (CODA). The first 2 years mainly include basic biomedical sciences (such as but not limited to biochemistry, physiology, embryology, histology, anatomy, and dental anatomy), while 3rd year mainly focuses on preclinical and dental subjects as (oral pathology, oral radiology, preclinical restorative dentistry). All of the first 3 years include lectures and laboratory sessions. The introduction of primary care clinics take place in the 4th year of the dental program and continues to comprehensive care practice (CCP) and comprehensive care clinics (CCC) in the 5th and 6th years, respectively. Attendance is considered mandatory as reflected in the University policy including KAU-FD.

Due to its potential impact on the dental educational system, better understanding of this phenomenon and the underlying causes became a task for educators in the field. Therefore, the aim of this study was to investigate dental students’ attitude in different dental years towards classroom attendance at KAU-FD, Jeddah, Saudi Arabia. To our knowledge, this is the first study to investigate dental students’ attendance pattern and its impact on the dental educational process.

## Materials and methods

A human research ethical approval was obtained through KAU-FD prior to launching the study. This was a cross-sectional study to survey all second to sixth year dental students actively enrolled at KAU-FD for their perception and attitude toward attending classroom lectures during the period from January 2019 to June 2019. The survey included fourteen questions on age, gender, marital status, number of children, average travel time to KAU-FD, current dental year, most recent GPA, student’s perspective and behavior toward classroom lectures. The study aimed to investigates students’ opinion on attendance, factors influencing their decision to do so and we also attempted to correlate students’ attendance with their academic performance using self-reported data as the study was anonymous in nature. The detailed questions of the survey are included in a Additional file 1.

Prior to distribution among participants, validation of the survey questions was completed through face validity by field experts to evaluate the ability to capture intended aims and check for errors such as unclear language and leading or distractive statements. The survey was then tested through enrolment of 10 dental students and collection of answers and feedback. Collected data were analyzed and used to modify 8 questions in total [8]. The survey cover-page included a consent form and a brief description of the study aim and design, possible benefits, and student assurance for anonymity and confidentiality. A hard copy of the survey was distributed by co investigators to study subjects from each level to ensure all students received and completed the survey only once. In order to enrol the maximum number of subjects, this survey was distributed and collected by the study co-investigators to students following mid-term exams based on accessibility.

At the end of the study, data were extracted and analysed using SPSS version 18.0 (SPSS Inc., Chicago, IL, USA) for statistical significance. Non-numerical descriptive data were presented as frequencies and percentages. Potential association between participants’ responses and gender, year level, or grade average were explored with chi-square test and significance level set at 0.001.

## Results

### Response rate and students’ demographics

A total of 893 full-time undergraduate dental students at KAU-FD were eligible to participate in this study in which 678 (75.92%) consented and completed the survey from all five dental levels during the period from January 2019 to June 2019. There was a total of 299 males (44.1%) and 379 female students (55.9%) with a mean age of 21 (range 18–24). Of all participants, twenty-three students (3.4%) were married, and eleven students (1.5%) had children. A total of 371 students (55.5%) had a GPA of 4.50–5.00 while 251 students (37.6%) had a GPA of 3.75–4.49. In all, study results were grouped into four categories affecting attendance, transportations time, students’ academic attitude, reasons behind absenteeism, and finally the subject type. A detailed demographics of the study participants and letter grades are described in Table 1.

**Table 1:** Demographics of study participants

Demographics	Categories	N (%)
Gender	Male	299 (44.1%)
	Female	379 (55.9%)
Marital Status	Single	652 (96.6%)
	Married	23 (3.4%)
Number of Children	None	661 (98.4%)
	One	7 (1.0%)
	Two	1 (0.1%)
	More than two	3 (0.4%)
Academic Average	< 3.74 (C-D)	47 (6.9%)
	3.75–4.49 (C-B)	251 (37.6%)
	4.50–5.00 (A-B)	371 (55.5%)
Academic Year	2nd	180 (26.6%)
	3rd	125 (18.5%)
	4th	90 (13.3%)
	5th	169 (25.0%)
	6th	113 (16.7%)

### Transportation time and classroom attendance

In order to understand the impact of transportation time on students’ punctuality, each participant was asked for their average travel time to KAU-FD. More than half of all students (51.4%) would need thirty to 60 min in transportation time to reach the dental school. At the same time, 16.8% would spend more than 60 min in transportation time to reach the dental school (Table 2).

**Table 2:** Students’ academic attitude towards classroom attendance

		Total	Gender		Academic year					Academic average		
			Male	Female	2nd	3rd	4th	5th	6th	< 3.75	3.75–4.49	4.50–5.00
How much time on average do you need to reach dental school	N	667	667		666					658		
	< 30 min	212 (31.8%)	122 (41.1%)	90 (24.3%)	62 (35.2%)	39 (32.8%)	30 (33.7%)	38 (22.5%)	43 (38.1%)	21 (45.7%)	71 (28.5%)	118 (32.5%)
	30–60 min	343 (51.4%)	131 (44.1%)	212 (57.3%)	83 (47.2%)	65 (54.6%)	46 (51.7%)	91 (53.8%)	57 (50.4%)	18 (39.1%)	132 (53.0%)	187 (51.5%)
	> 60 min	112 (16.8%)	44 (14.8%)	68 (18.4%)	31 (17.6%)	15 (12.6%)	13 (14.6%)	40 (23.7%)	13 (11.5%)	7 (15.2%)	46 (18.5%)	58 (16.0%)
	*p*-value		*p* < 0.001^¥^*		*p* = 0.042^¥^* *p*=					*p* = 0.218^¥^		
Do you think classes should be optional for students?	N	664	664		668					655		
	No	160 (24.1%)	66 (22.3%)	94 (25.5%)	34 (19.4%)	30 (25.2%)	26 (28.9%)	44 (26.5%)	25 (22.1%)	7 (15.9%)	58 (23.2%)	93 (25.9%)
	Yes	504 (75.9%)	230 (77.7%)	274 (74.5%)	141 (80.6%)	89 (74.8%)	64 (71.1%)	122 (73.5%)	88 (77.9%)	39 (84.8%)	192 (76.8%)	266 (74.1%)
	*p*-value		*p* = 0.331^¥^		*p* = 0.396^¥^					*p* = 0.255^¥^		
Do you think attending classes have a positive influence on students’ academic performance?	N	665	665		664					656		
	No	161 (24.2%)	96 (32.4%)	65 (17.6%)	70 (39.8%)	20 (16.5%)	13 (14.4%)	27 (16.5%)	31 (27.4%)	15 (34.1%)	55 (22.1%)	90 (24.9%)
	Yes	504 (75.8%)	200 (67.6%)	304 (82.4%)	106 (60.2%)	101 (83.5%)	77 (85.6%)	137 (83.5%)	82 (72.6%)	31 (67.4%)	194 (77.9%)	271 (75.1%)
	*p*-value		*p* < 0.001^¥^*		*p* < 0.001^¥^*					*p* = 0.293^¥^		
How many times per month on average do you skip classes?	N	671	671		670					661		
	0–2 times	297 (44.3%)	89 (30.2%)	208 (55.3%)	70 (39.1%)	66 (53.7%)	45 (50.0%)	66 (39.5%)	50 (45.0%)	13 (28.9%)	98 (39.5%)	181 (49.2%)
	3–4 times	204 (30.4%)	90 (30.5%)	114 (30.3%)	53 (29.6%)	38 (30.9%)	19 (21.1%)	60 (35.9%)	33 (29.7%)	13 (28.9%)	85 (34.3%)	104 (28.3%)
	5–6 times	80 (11.9%)	48 (16.3%)	32 (8.5%)	14 (7.8%)	11 (8.9%)	13 (14.4%)	26 (15.6%)	16 (14.4%)	10 (22.2%)	34 (13.7%)	34 (9.2%)
	More than 6 times	90 (13.4%)	68 (23.1%)	22 (5.9%)	42 (23.5%)	8 (6.5%)	13 (14.4%)	15 (9.0%)	12 (10.8%)	9 (20.0%)	31 (12.5%)	49 (13.3%)
	*p*-value		*p* < 0.001^¥^*		*p* < 0.001^¥^*					*p* = 0.014^¥^*		
If KAUFD installs video recording devices in classrooms to record live lectures and make it available, are you:	N	644	644		643					634		
	Less likely to skip or be late to a lecture	147 (22.8%)	57 (20.1%)	90 (25.0%)	41 (24.1%)	25 (20.7%)	27 (31.4%)	32 (20.4%)	21 (19.3%)	9 (20.5%)	53 (22.4%)	83 (23.5%)
	No difference	312 (48.4%)	138 (48.6%)	174 (48.3%)	88 (51.8%)	70 (57.9%)	31 (36.0%)	77 (49.0%)	46 (42.2%)	24 (54.5%)	103 (43.5%)	178 (50.4%)
	More likely to skip or be late to a lecture	185 (28.7%)	89 (31.3%)	96 (26.7%)	41 (24.1%)	26 (21.5%)	28 (32.6%)	48 (30.6%)	42 (38.5%)	11 (25.0%)	81 (34.2%)	92 (26.1%)
	*p*-value		*p* = 0.235^¥^		*p* = 0.02^¥^*					*p* = 0.238^¥^		

### Students’ attitude towards classroom attendance

More than three quarters of participants (75.9%) were more in favour of optional classroom attendance. However, when asked about benefits of attending classroom lectures, 75.8% of participants strongly believed that classroom lectures have a positive influence on their academic performance which was more significant in female students (82.4% vs 67.6%; *p* < 0.001). Out of all levels, second year students were the least (60.2%) to assume positive gain of class attendance on academic performance compared to students in higher levels (*p* < 0.001) (Table 2).

In terms of frequency of dental students not attending classroom lectures, 44.3% of students were more likely to skip two classes or less per month; 30.4% of students would skip three to four classes per month, and 25.3% of students would skip classroom lectures five times or more per month. More than half of female students (55.3%) skipped two or less classes per month compared to male students (30.2%); while 39.4% of male and 14.4% female students missed more than 5 classes per month (*p* < 0.001). Further analysis of the data demonstrated that 42.2% of under-achievers with average academic performance of C or D were more likely to skip five classroom lectures or more compared to A-students (22.5%) and B-students (16.2%) (Table 2). When asked if the dental school would provide video recording of all lectures to be accessed at a later and more convenient time, 28.7% of students were likely to continue skipping or be late to a lecture. However, 48.4% of students were neutral, and 22.8% of participating students were likely to be more punctual and attend more classroom lectures even with access to lectures video recordings.

### Reasons behind students’ absence or late attendance to lectures

To better understand students’ rationale towards attending or skipping classroom lectures, participants were asked to list the top three reasons behind their decision to skip a specific lecture. Lecture time was reported as the main factor to influence students’ decision to attend a specific lecture. Out of all students, 47.9% were more likely to arrive late or skip the 8 a.m. lecture which was more frequent in males compared to females (56 and 41.2% respectively; *p* < 0.001). Furthermore, under-achievers (69.6%) were more likely to skip the morning lecture compared to A-students (42.6%) and B-students (52.2%) (Table 3). Preparation for exams was the second most common reason affecting students’ classroom attendance (42%) in both genders. This trend was more evident in A-students (47.2%) compared to B-students (37.8%) and underachievers (32.6%; *p* = 0.025) as well as in second year (50.0%), third year (47.2%), and fourth year dental students (43.3%; *p* = 0.041) (Table 3). Lecturer’s presentation skills and delivery of information were reported as the third most common reason to influence students’ attendance. In total, 41.9% of students likely to skip classes given by lecturers known to have less than average lecturing skills. No trends or associations were found between lecturer’s presentation skills and gender, level, or grade average. A completed list of reported reasons behind students’ absence or late attendance to classroom lectures can be found in (Table 3).

**Table 3:** Reasons behind students’ absence or late attendance to classroom lectures

Reasons for why you would be late or absent from a lecture		Total	Gender		Academic year					Academic average		
			Male	Female	2nd	3rd	4th	5th	6th	< 3.75	3.75–4.49	4.50–5.00
The lecture is before or after an examination	N	678	678		677					668		
	No	387 (57.1%)	170 (56.9%)	217 (57.3%)	90 (50.0%)	66 (52.8%)	51 (56.7%)	104 (61.5%)	75 (66.4%)	31 (67.4%)	156 (62.2%)	196 (52.8%)
	Yes	291 (42.9%)	129 (43.1%)	162 (42.7%)	90 (50.0%)	59 (47.2%)	39 (43.3%)	65 (38.5%)	38 (33.6%)	15 (32.6%)	95 (37.8%)	175 (47.2%)
	*p*-value		*p* = 0.917^¥^		*p* = 0.041^¥^*					*p* = 0.025^¥^*		
Early morning class (8–9 am)	N	678	678		677					668		
	No	353 (52.1%)	130 (43.5%)	223 (58.8%)	107 (59.4%)	83 (66.4%)	50 (55.6%)	70 (41.4%)	43 (38.1%)	14 (30.4%)	120 (47.8%)	213 (57.4%)
	Yes	325 (47.9%)	169 (56.5%)	156 (41.2%)	73 (40.6%)	42 (33.6%)	40 (44.4%)	99 (58.6%)	70 (61.9%)	32 (69.6%)	131 (52.2%)	158 (42.6%)
	*p*-value		*p* < 0.001^¥^*		*p* < 0.001^¥^*					*p* < 0.001^¥^*		
Late class (3–5 pm)	N	678	678		677					668		
	No	465 (68.6%)	212 (70.9%)	253 (66.8%)	136 (75.6%)	82 (65.6%)	51 (56.7%)	110 (65.1%)	85 (75.2%)	29 (63.0%)	161 (64.1%)	268 (72.2%)
	Yes	213 (31.4%)	87 (29.1%)	126 (33.2%)	44 (24.4%)	43 (34.4%)	39 (43.3%)	59 (34.9%)	28 (24.8%)	17 (37.0%)	90 (35.9%)	103 (27.8%)
	*p*-value		*p* = 0.248^¥^		*p* = 0.008^¥^*					*p* = 0.072^¥^		
If I have two or more-hour break before or after class	N	678	678		677					668		
	No	570 (84.1%)	243 (81.3%)	327 (86.3%)	146 (81.1%)	92 (73.6%)	80 (88.9%)	150 (88.8%)	101 (89.4%)	40 (87.0%)	210 (83.7%)	310 (83.6%)
	Yes	108 (15.9%)	56 (18.7%)	52 (13.7%)	34 (18.9%)	33 (26.4%)	10 (11.1%)	19 (11.2%)	12 (10.6%)	6 (13.0%)	41 (16.3%)	61 (16.4%)
	*p*-value		*p* = 0.077^¥^		*p* = 0.001^¥^*					*p* = 0.837^¥^		
Lecture material is available on Blackboard, video, or voice recording or another source	N	677	677		676					667		
	No	643 (95.0%)	289 (96.7%)	354 (93.7%)	163 (91.1%)	116 (92.8%)	86 (95.6%)	165 (97.6%)	112 (99.1%)	45 (97.8%)	244 (97.2%)	345 (93.2%)
	Yes	34 (5.0%)	10 (3.3%)	24 (6.3%)	16 (8.9%)	9 (7.2%)	4 (4.4%)	4 (2.4%)	1 (0.9%)	1 (2.2%)	7 (2.8%)	25 (6.8%)
	*p*-value		*p* = 0.075^¥^		*p* = 0.006^F^*					*p* = 0.064^F^		
The lecturer is below expectation (e.g. read directly from slides, lack clarity and organisation)	N	678	678		677					668		
	No	394 (58.1%)	181 (60.5%)	213 (56.2%)	117 (65.0%)	69 (55.2%)	52 (57.8%)	95 (56.2%)	60 (53.1%)	24 (52.2%)	158 (62.9%)	205 (55.3%)
	Yes	284 (41.9%)	118 (39.5%)	166 (43.8%)	63 (35.0%)	56 (44.8%)	38 (42.2%)	74 (43.8%)	53 (46.9%)	22 (47.8%)	93 (37.1%)	166 (44.7%)
	*p*-value		*p* = 0.265^¥^		*p* = 0.252^¥^					*p* = 0.116^¥^		
The lecturer is not strict about student attendance	N	678	678		677					668		
	No	627 (92.5%)	273 (91.3%)	354 (93.4%)	172 (95.6%)	118 (94.4%)	78 (86.7%)	158 (93.5%)	100 (88.5%)	43 (93.5%)	226 (90.0%)	348 (93.8%)
	Yes	51 (7.5%)	29 (8.7%)	25 (6.6%)	8 (4.4%)	7 (5.6%)	12 (13.3%)	11 (6.5%)	13 (11.5%)	3 (6.5%)	25 (10.0%)	23 (6.2%)
	*p*-value		*p* = 0.303^¥^		*p* = 0.036^¥^*					*p* = 0.214^¥^		
I am not interested in a specific topic	N	678	678		677					668		
	No	567 (83.6%)	246 (82.3%)	321 (84.7%)	155 (86.1%)	116 (92.8%)	67 (74.4%)	142 (84.0%)	87 (77.0%)	40 (87.0%)	210 (83.7%)	310 (83.6%)
	Yes	111 (16.4%)	53 (17.7%)	58 (15.3%)	25 (13.9%)	9 (7.2%)	23 (25.6%)	27 (16.0%)	26 (23.0%)	6 (13.0%)	41 (16.3%)	61 (16.4%)
	*p*-value		*p* = 0.397^¥^		*p* = 0.001^¥^*					*p* = 0.903 ^F^		
I can learn the subject of the day better by spending the same time studying at home	N	678	678		677					668		
	No	476 (70.2%)	215 (71.9%)	261 (68.9%)	100 (55.6%)	84 (67.2%)	62 (68.9%)	137 (81.1%)	93 (82.3%)	36 (78.3%)	190 (75.7%)	242 (65.2%)
	Yes	202 (29.8%)	84 (28.1%)	118 (31.1%)	80 (44.4%)	41 (32.8%)	28 (31.1%)	32 (18.9%)	20 (17.7%)	10 (21.7%)	61 (24.3%)	129 (34.8%)
	*p*-value		*p* = 0.390^¥^		*p* < 0.001^¥^*					*p* = 0.009^¥^*		
I don’t wake up early	N	678	678		677					668		
	No	625 (92.2%)	267 (89.3%)	358 (94.5%)	166 (92.2%)	121 (96.8%)	83 (92.2%)	151 (89.3%)	103 (91.2%)	40 (87.0%)	226 (90.0%)	350 (94.3%)
	Yes	53 (7.8%)	32 (10.7%)	21 (5.5%)	14 (7.8%)	4 (3.2%)	7 (7.8%)	18 (10.7%)	10 (8.8%)	6 (13.0%)	25 (10.0%)	21 (5.7%)
	*p*-value		*p* = 0.013^¥^*		*p* = 0.181 ^F^					*p* = 0.056^¥^		
Difficulty with transportation	N	678	678		677					668		
	No	611 (90.1%)	271 (90.6%)	340 (89.7%)	167 (92.8%)	110 (88.0%)	81 (90.0%)	147 (87.0%)	105 (92.9%)	44 (95.7%)	218 (86.9%)	342 (92.2%)
	Yes	67 (9.9%)	28 (9.4%)	39 (10.3%)	13 (7.2%)	15 (12.0%)	9 (10.0%)	22 (13.0%)	8 (7.1%)	2 (4.3%)	33 (13.1%)	29 (7.8%)
	*p*-value		*p* = 0.688^¥^		*p* = 0.296^¥^					*p* = 0.048 ^F^*		
I don’t feel attending classes benefits me	N	678	678		677					668		
	No	594 (87.6%)	258 (86.3%)	336 (88.7%)	144 (80.0%)	108 (86.4%)	82 (91.1%)	149 (88.2%)	110 (97.3%)	39 (84.8%)	227 (90.4%)	319 (86.0%)
	Yes	84 (12.4%)	41 (13.7%)	43 (11.3%)	36 (20.0%)	17 (13.6%)	8 (8.9%)	20 (11.8%)	3 (2.7%)	7 (15.2%)	24 (9.6%)	52 (14.0%)
	*p*-value		*p* = 0.353^¥^		*p* < 0.001 ^F^*					*p* = 0.214^¥^		

^¥^ Chi-square test

^F^ Fisher Exact test

* Statistically significant *p* < 0.05

### The influence of subject type on classroom attendance

To understand the impact of lecture subject on classroom attendance, study participants were asked to rank the top three dental subjects they were more likely to skip or be late for their lectures. Among second year students, non-dental basic science subjects were the most likely to be skipped as the following: histology and embryology (69.4%), biochemistry (60.0%) and gross anatomy (56.1%) (Table 4). Among third year dental students, basic science subjects were also the least popular with microbiology class being the most likely to be skipped (58.4%) followed by general pathology (25.0%). For fourth year dental students, general medicine was reported to have the highest percentage of absenteeism (32.2%) followed by removable prosthodontics (17.8%). Among fifth year level students, pharmacology class had an absence rate of 41.4%, followed by removable prosthodontics class (40.8%). For sixth year students, only three subjects were likely to be skipped including oral surgery (8.0%), orthodontics (6.2%) and pedodontics (5.3%) albeit at much lower percentages in comparison to the aforementioned years.

**Table 4:** Influence of type of subject on students’ classroom attendance

Academic year	Subjects you are more likely to skip or be late for their lectures		Total	Gender		Academic average		
				Male	Female	< 3.75	3.75–4.49	4.50–5.00
2nd year *n* = 180	Gross Anatomy	No	79 (43.9%)	30 (39.5%)	49 (47.1%)	0 (0.0%)	1 (33.3%)	78 (44.6%)
		Yes	101 (56.1%)	46 (60.5%)	55 (52.9%)	1 (100.0%)	2 (66.6%)	97 (55.4%)
	*p*-value			*p* = 0.362^¥^		*p* = 1.00 ^F^		
	Biochemistry	No	72 (40.0%)	11 (14.5%)	61 (58.7%)	0 (0.0%)	1 (33.3%)	71 (40.6%)
		Yes	108 (60.0%)	65 (85.5%)	43 (41.3%)	1 (100.0%)	2 (66.7%)	104 (59.4%)
	*p*-value			*p* < 0.001^¥^*		*p* = 1.00 ^F^		
	Histology and Embryology	No	55 (30.6%)	19 (25.0%)	36 (34.6%)	0 (0.0%)	2 (66.7%)	53 (30.0%)
		Yes	125 (69.4%)	57 (75.0%)	68 (65.4%)	1 (100.0%)	1 (33.3%)	122 (69.7%)
	*p*-value			*p* = 0.192^¥^		*p* = 0.464 ^F^		
	Dental Anatomy	No	155 (86.1%)	68 (89.5%)	87 (83.7%)	1 (100.0%)	2 (66.7%)	151 (86.3%)
		Yes	25 (13.9%)	8 (10.5%)	17 (16.3%)	0 (0.0%)	1 (33.3%)	24 (13.7%)
	*p*-value			*p* = 0.285^¥^		*p* = 0.455 ^F^		
	Biomaterials	No	161 (89.4%)	57 (75.0%)	104 (100.0%)	1 (100.0%)	3 (100.0%)	156 (89.1%)
		Yes	19 (10.6%)	19 (25.0%)	0 (0.0%)	0 (0.0%)	0 (0.0%)	19 (10.9%)
	*p*-value			*p* < 0.001 ^F^*		*p* = 1.00 ^F^		
3rd year *n* = 125	Operative Dentistry	No	124 (99.2%)	59 (100.0%)	65 (98.5%)	4 (100.0%)	49 (100.0%)	69 (98.6%)
		Yes	1 (0.8%)	0 (0.0%)	1 (1.5%)	0 (0.0%)	0 (0.0%)	1 (1.4%)
	*p*-value			*p* = 1.00 ^F^		*p* = 1.00 ^F^		
	Biomaterials	No	113 (90.4%)	55 (93.2%)	58 (87.9%)	3 (75.0%)	46 (93.9%)	62 (88.6%)
		Yes	12 (9.6%)	4 (6.8%)	8 (12.1%)	1 (25.0%)	3 (6.1%)	8 (11.4%)
	*p*-value			*p* = 0.373 ^F^		*p* = 0.279 ^F^		
	Microbiology	No	52 (41.6%)	14 (23.7%)	38 (57.6%)	2 (50.0%)	21 (42.9%)	28 (40.0%)
		Yes	73 (58.4%)	45 (76.3%)	28 (42.4%)	2 (50.0%)	28 (57.1%)	42 (60.0%)
	*p*-value			*p* < 0.001^¥^*		*p* = 0.946 ^F^		
	Oral Histology	No	103 (82.4%)	49 (83.1%)	54 (81.8%)	3 (75.0%)	38 (77.6%)	60 (85.7%)
		Yes	22 (17.6%)	10 (16.9%)	12 (18.2%)	1 (25.0%)	11 (22.4%)	10 (14.3%)
	*p*-value			*p* = 0.857^¥^		*p* = 0.383 ^F^		
	Oral Pathology	No	117 (93.6%)	56 (94.9%)	61 (92.4%)	4 (100.0%)	47 (95.9%)	64 (91.4%)
		Yes	8 (6.4%)	3 (5.1%)	5 (7.6%)	0 (0.0%)	2 (4.1%)	6 (8.6%)
	*p*-value			*p* = 0.721 ^F^		*p* = 0.595 ^F^		
	Oral Radiology	No	114 (91.2%)	51 (86.4%)	63 (95.5%)	4 (100.0%)	41 (83.7%)	67 (95.7%)
		Yes	11 (8.8%)	8 (13.6%)	3 (4.5%)	0 (0.0%)	8 (16.3%)	3 (4.3%)
	*p*-value			*p* = 0.113 ^F^		*p* = 0.098 ^F^		
	General Pathology	No	100 (80.0%)	46 (78.0%)	54 (81.8%)	4 (100.0%)	35 (71.4%)	59 (84.3%)
		Yes	25 (25.0%)	13 (22.0%)	12 (18.2%)	0 (0.0%)	14 (28.6%)	11 (15.7%)
	*p*-value			*p* = 0.657 ^F^		*p* = 0.168 ^F^		
	Pharmacology	No	112 (89.6%)	55 (93.2%)	57 (86.4%)	3 (75.0%)	45 (91.8%)	62 (88.6%)
		Yes	13 (10.4%)	4 (6.8%)	9 (13.6%)	1 (25.0%)	4 (8.2%)	8 (11.4%)
	*p*-value			*p* = 0.251 ^F^		*p* = 0.608 ^F^		
	Pain Control	No	125 (100.0%)	59 (100.0%)	59 (100.0%)	4 (100.0%)	49 (100.0%)	70 (100.0%)
		Yes	0 (0.0%)	0 (0.0%)	0 (0.0%)	0 (0.0%)	0 (0.0%)	0 (0.0%)
Academic year	Subjects you are more likely to skip or be late for their lectures		Total	Gender		Academic average		
				Male	Female	< 3.75	3.75–4.49	4.50–5.00
4th year *n* = 90	Operative Dentistry	No	86 (95.6%)	36 (97.3%)	50 (94.3%)	11 (100.0%)	46 (93.9%)	28 (96.6%)
		Yes	4 (4.4%)	1 (2.7%)	3 (5.7%)	0 (0.0%)	3 (6.1%)	1 (3.4%)
	*p*-value			*p* = 0.641 ^F^		*p* = 1.00 ^F^		
	Endodontics	No	82 (91.1%)	30 (81.1%)	52 (98.1%)	10 (90.9%)	45 (91.8%)	27 (93.1%)
		Yes	8 (8.9%)	7 (18.9%)	1 (1.9%)	1 (9.1%)	4 (8.2%)	2 (6.9%)
	*p*-value			*p* = 0.008 ^F^*		*p* = 1.00 ^F^		
	General Medicine	No	61 (67.8%)	26 (70.3%)	35 (66.0%)	9 (81.8%)	33 (67.3%)	18 (62.1%)
		Yes	29 (32.2%)	11 (29.6%)	18 (34.0%)	2 18.2(%)	16 (32.7%)	11 (37.9%)
	*p*-value			*p* = 0.672^¥^		*p* = 0.561 ^F^		
	Oral Biology	No	88 (97.8%)	35 (94.6%)	53 (100.0%)	10 (90.9%)	48 (98.0%)	29 (100.0%)
		Yes	2 (2.2%)	2 (5.4%)	0 (0.0%)	1 (9.1%)	1 (2.0%)	0 (0.0%)
	*p*-value			*p* = 0.166 ^F^		*p* = 0.337 ^F^		
	Periodontics	No	82 (91.1%)	34 (91.9%)	48 (90.6%)	11 (100.0%)	46 (93.9%)	24 (82.8%)
		Yes	8 (8.9%)	3 (8.1%)	5 (9.4%)	0 (0.0%)	3 (6.1%)	5 (17.2%)
	*p*-value			*p* = 1.00 ^F^		*p* = 0.179 ^F^		
	Oral Radiology	No	81 (90.0%)	32 (86.5%)	49 (92.5%)	10 (90.9%)	44 (89.8%)	26 (89.7%)
		Yes	9 (10.0%)	5 (13.5%)	4 (7.5%)	1 (9.1%)	5 (10.2%)	3 (10.3%)
	*p*-value			*p* = 0.479 ^F^		*p* = 1.00 ^F^		
	Removable Prosthodontics	No	74 (82.2%)	29 (78.4%)	45 (84.9%)	9 (81.8%)	40 (81.6%)	24 (82.8%)
		Yes	16 (17.8%)	8 (21.6%)	8 (15.1%)	2 (18.2%)	9 (18.4%)	5 (17.2%)
	*p*-value			*p* = 0.576^¥^		*p* = 1.00 ^F^		
	Fixed Prosthodontics	No	80 (88.9%)	34 (91.9%)	46 (86.8%)	10 (90.9%)	44 (89.8%)	25 (86.2%)
		Yes	10 (11.1%)	3 (8.1%)	7 (13.2%)	1 (9.1%)	5 (10.2%)	4 (13.8%)
	*p*-value			*p* = 0.516 ^F^		*p* = 0.889 ^F^		
	Ethics and Law	No	85 (94.4%)	34 (91.9%)	51 (96.2%)	10 (90.9%)	45 (91.8%)	29 (100.0%)
		Yes	5 (5.6%)	3 (8.1%)	2 (3.8%)	1 (9.1%)	4 (8.2%)	0 (0.0%)
	*p*-value			*p* = 0.398 ^F^		*p* = 0.30 ^F^		
	Biostatistics	No	85 (94.4%)	34 (91.9%)	51 (96.2%)	10 (90.9%)	45 (91.8%)	29 (100.0%)
		Yes	5 (5.6%)	3 (8.1%)	2 (3.8%)	1 (9.1%)	4 (8.2%)	0 (0.0%)
	*p*-value			*p* = 0.398 ^F^		*p* = 0.30 ^F^		
	General Surgery	No	82 (91.1%)	32 (86.5%)	50 (94.3%)	10 (90.9%)	42 (85.7%)	29 (100.0%)
		Yes	8 (8.9%)	5 (13.5%)	3 (5.7%)	1 (9.1%)	7 14.3(%)	0 (0.0%)
	*p*-value			*p* = 0.266 ^F^		*p* = 0.073 ^F^		

^¥^ Chi-square test

^F^ Fisher Exact test

* Statistically significant *p* < 0.05

## Discussion

For the past several years, the international demand for dental schools’ admission has been on the rise with an extensive and challenging selection process [9]. Consequently, all students are expected to maintain an acceptable level of classroom attendance to achieve academic excellence as candidates for dental degrees [10]. However, a certain number of students may not be as passionate as expected with higher tendency to skip classroom lectures for different reasons which has been observed among dental students for the past several years [1, 2, 10, 11]. Although classroom lectures could be attained by other sources of online sessions, attendance regardless of the mode of delivery is nonetheless paramount [1]. It plays a crucial role in the acquisition of knowledge especially pertaining to professional training in health sciences [1]. Considering dentistry as a field which relies on students’ ability to combine and integrate knowledge with clinical skills, the current study was designed to understand student’s attitude and behavior toward classroom lectures. In addition, it explores an action plan to reduce the impact of lack of classroom attendance on educational outcomes.

Lecture absenteeism is a controversial debate with regard to its connection to academic performance. Numerous studies have reported on the causal relationship between non-attendance and academic achievement [1, 5]. Looking at the current data, several factors had an impact on students’ attitude towards classroom attendance. Class timing had a major impact on overall classroom attendance. Early lectures were those most frequently missed as reported in numerous other studies [4, 12, 13]. This could be a result of the students’ lifestyle as they tend to stay up late during the week resulting in lack of sufficient sleep. Another compounding factor could be commuting in heavy traffic. Exam preparation ranked second as a main reason for missing lectures. This finding may be related to poor student planning or lack of interest. Lecturers’ overall skills were reported as the third most influential factor on students’ attendance. Criteria for unsatisfactory lecture styles included less organized slides, long lectures and non- interactive sessions. Considering the reported lecture attendees’ attention span to be around 20 min, lecturers with more engaging teaching skills are more likely to be popular among students [2].

Another factor identified in this study with significant impact on classroom attendance was student transportation and time management [2]. More than half of participants had to spend between thirty and 60 min to reach the dental school, while some spent over 1 h on the road. Considering Jeddah’s busy and heavy traffic city, these numbers fall within average commuting times. A study conducted at the University of California-Davis School of Medicine reported that 24% of second year medical students enrolled in a dermatology course have ranked travel time as a major factor for classroom absence [13]. Considering travel time variability, which may be based on factors like city road plan, availability of public transportation and distance between student’s home and dental school, students are expected to plan their trip to school ahead of time and account for possible traffic delays in order to make it on time to classroom lectures.

The available literature has reported other several factors provided by students to influence classroom attendance which were more personal and included the following: social expectation; did not want to disappoint the speaker; opportunity to interact with the speaker; opportunity to socialise with classmates; because they had paid the tuition; and learning well in a classroom-type setting as well as interesting lecture topic and reputation of speaker which were also reported in the current cohort of participants [4, 7, 13] On the other hand, close monitoring by faculty members for attendance in classrooms, better teaching skills, topics more relevant to dentistry and in-class clinical and lab tips which may affects students’ academic performance were some of the factors which would encourage students’ attendance and warrant further evaluation.

Data on the relationship between students’ self-reported GPA and class attendance was included in the current study. A-students were more likely to attend classroom lectures compared to under-achievers with average academic performance of C or D who were more likely to skip five classroom lectures or more. Hyde et al. reported similar data, where students who attended 80–100% of their scheduled lectures had better academic performance compared to their peers [14]. Even with existing interest to attend classroom lectures, A-students were more likely to miss classes to prepare for exams compared to C or D students. This finding is comparable to the current literature in which preparation for exams was reported to be a frequent reason for missing a classroom lecture (58%) [4, 7].

Whether lecture attendance should be optional or not has been a recent active debate across dental schools [15]. Several international dental schools have shifted toward optional attendance, and the rest continue to mandate students’ attendance in classroom lectures for all levels including schools in Saudi Arabia (unpublished data). Considering the current COVID19 outbreak, many educational institutes around the globe were forced to shift toward virtual learning changing the paradigm of students’ education [16]. Historically, conventional education system supported students’ physical attendance in classroom lectures with the purpose to gain the maximum level of information via interactive sessions with lecturers. In addition, providing an opportunity to communicate with the academic staff for further explanation of less clear information [15]. Even in situations where lectures are not particularly well structured, education specialists believed students would still gain basic information on a particular subject [17]. On the other hand, optional classroom attendance provides more flexibility and additional free time for other academic activities such as research or improving clinical skills provided that lecture recordings are available for students.

The available literature includes a mixture of data on the impact of attendance on academic performance. A series of recent studies have demonstrated classroom attendance predicts academic outcomes and future achievements. In addition, students with higher classroom attendance demonstrated better competencies and higher grades in a given academic year [18]. This observation was supported by a study which included civil engineering students at University College of Dublin and demonstrated that students with higher lecture attendance rates clearly outperformed those with poor attendance records [19]. In addition, passing grades of 40% were attained at relatively low attendance levels (< 20% attendance). Contrarily, other studies failed to report a correlation between both classroom attendance and academic performance [3, 4, 20, 21]. A certain group of students reported by Hyde et al. who did not attend classroom lectures regularly, were found to perform well in exams. These students are likely to better perform away from the classroom crowd to overcome factors such as social anxiety and fear. For these cases, the authors advised against mandatory classroom attendance as it would adversely affect the students’ overall performance [14]. Simultaneously, psychiatric help may be needed to support students who need to overcome their social challenges. However, student motivation and improving personal skills were reported to have a more significant impact on students’ performance compared to classroom attendance [22].

The availability of the lecture material through various resources and its link to students’ attendance has been a topic of discussion in the academic community. In the current study, only 5% of the participants reported to be less motivated to attend classroom lectures if the lecture material was available via colleagues, online or other sources. This finding is partly in line with a similar study conducted at Tufts Dental School (Boston, MA) which found a weak correlation between attendance of pre-clinical courses in classrooms and the availability of learning material online [3]. However, 35% of students enrolled in a dermatology course indicated they were less interested in attending classroom lectures if the material was made available online [13]. Another study reported 27% of respondents expressing an interest in a total of 18 lectures’ audio and video recordings which were available as online material and found them useful to help with exam preparation [23].

By analysing the current data and the reported factors influencing classroom attendance, the dental education community may be able to modify current teaching strategies to implement innovative methods to encourage better attendance for the newer generation of dental students. This in turn would foster a better learning environment, engaging students in interactive discussions and promoting better attendance [24]. Moreover, today’s generation of students use digital platforms as part of their daily lives. Thus, incorporating this approach into the learning process at dental schools and providing online educational material created by dental school faculty to be available for students as a reference are of the utmost importance. This would provide better and supervised resources for students to guarantee quality of delivered information.

This study has several limitations. First, the nature of the study provides a potential for under-reporting classroom attendance. In general, it is vital to separate self-reported attendance from actual attendance records to provide a more accurate overview as students are more inclined to report higher attendance levels to avoid any academic consequences [25]. Although this study relies on self-reporting, the overall number of attending students were comparable to what has been reported in the Arabian Gulf region [4]. In addition, participants were informed about the anonymous nature of the study prior to the distribution of the survey ensuring they felt safe resulting in a more accurate data collection. Second, the current study was conducted in a single dental institution, and it would be challenging to generalize these results to all dental schools in Saudi Arabia. However, it does provide an overview of a large group of students’ attitudes toward classroom attendance. On the other hand, this study recruited dental student from one of the largest schools in Saudi Arabia. Hence, factors discussed in this study can be generalized overall population of Saudi Arabia. Additionally, it includes students’ self-reported data which is a major contributor in the educational process. Third, the current survey asked questions on lectures only excluding pre-clinical and clinical sessions which should be included in future studies.

## Conclusion

In general, our data provides a first look at students’ attitude and factors which may impact their classroom attendance. Future studies may explore potential solutions for classroom absence and propose modifications to the educational system to improve learning outcomes in the field of dentistry. In addition, implementation of more attractive, advanced technologies to replace traditional teaching methods should be explored to increase the level of student attendance.

## Supplementary Information

**Additional file 1.** Student attendance survey.

## Data Availability

The datasets used and/or analysed during the current study are available from the corresponding author on reasonable request.
