# Correction to: Why do students skip classroom lectures: A single dental school report

**DOI:** 10.1186/s12909-021-02866-7

**Published:** 2021-08-12

**Authors:** Waleed A. Alamoudi, Azza F. Alhelo, Soulafa A. Almazrooa, Osama M. Felemban, Nada O. Binmadi, Nada A. Alhindi, Sarah A. Ali, Sara K. Akeel, Sana A. Alhamed, Ghadah M. Mansour, Hani H. Mawardi

**Affiliations:** 1 Department of Oral Diagnostic Sciences, King Abdul-Aziz University- Faculty of Dentistry, Jeddah, Saudi Arabia; 2 Department of Pediatric Dentistry, King Abdulaziz University- Faculty of Dentistry, Jeddah, Saudi Arabia

**Correction to: BMC Medical Education 21, 388 (2021)**

**https://doi.org/10.1186/s12909-021-02824-3**

Following publication of the original article [1], due to a typesetting error, the authors were incorrectly affiliated.

The authors’ affiliations have been updated above and the original article [1] has been corrected.
