# Supplementary material for: Why do students skip classroom lectures: A single dental school report
Source: BMC Med Educ. 2021 Jul 20;21:388. doi: 10.1186/s12909-021-02824-3 (PMC8293555; doi:10.1186/s12909-021-02824-3)
Supplement: Supplementary file 1 — Additional file 1. Student attendance survey. [file 12909_2021_2824_MOESM1_ESM.pdf]

Student attendance survey

You are invited to participate in this survey on KAU-FD student attendance. This is a research project being conducted and it should take approximately 3 minutes to complete.

PARTICIPATION
Your participation in this survey is voluntary. You may refuse to take part in the research or exit the survey at any time without penalty. You are free to decline to answer any particular question you do not wish to answer for any reason.

BENEFITS
You will receive no direct benefits from participating in this research study. However, your responses may help us learn more about student attendance behavior at KAU-FD.

RISKS
There is the risk that you may find some of the questions to be sensitive.

CONFIDENTIALITY
Your survey answers will stored in a password protected electronic format. We are not collecting identifying information such as your name, email address. Therefore, your responses will remain anonymous. No one will be able to identify you or your answers, and no one will know whether or not you participated in the study.

CONTACT
If you have questions at any time about the study or the procedures, you may contact the research supervisor, Dr. Hani Mawardi via email at hmawardi@kau.edu.sa.

Please select your choice below. You may print a copy of this consent form for your records. Clicking on the “Agree” button indicates that:

- You have read the above information
- You voluntarily agree to participate
- You are 18 years of age or older

☐ Agree

☐ Disagree

Dental Students' Attendance: A Survey

1- **Age:**

☐18-20 ☐21-25 ☐26-30 ☐31-35

2- **Current Dental Year:**

☐2nd year ☐3rd year ☐4th year ☐5th year ☐6th year

3- **Gender:**

☐Male ☐Female

4- **Marital status:**

☐Single ☐Married ☐Divorced ☐Widowed

5- **Number of children:**

☐1 ☐2 ☐>2 ☐None

6- **Your academic average for the last year (GPA):**

☐<2.74 ☐2.75-3.74 ☐3.75-4.49 ☐4.50 -5.00

7- **How much time on average do you need to reach the dental school?**

☐<30 minutes ☐30-60 minutes ☐61-120 minutes ☐>120 minutes

8- **Do you think attending classes should be <u>optional</u> for students?**

☐Yes ☐No

9- **Do you think attendaning classes have a positive influence on students' performance?**

☐Yes ☐ No

10- **How many times per month on average do you <u>skip</u> a lecture?**

☐1-2 times ☐3-4 times ☐5-6 times ☐More than 6

11- **How many times per month on average you are late to a lecture?**

☐1-2 times ☐3-4 times ☐5-6 times ☐More than 6

12- **If KAU-FD installs video recording devices in classrooms to record live lectures and make it available, are you:**

☐More likely to skip or be late to a lecture ☐Less likely to skip or be late to a lecture

☐No difference

13- **What are the <u>top 3 subjects</u> you are more likely to skip or be late for their lectures**?

☐Anatomy ☐General Histology & Embryology ☐Biochemistry
☐Microbiology ☐General and Systemic Pathology ☐Dental anatomy
☐Oral Histology ☐Oral pathology ☐Oral Biology
☐Oral radiology ☐General medicine ☐General Surgery
☐Operative & Esthetic Dentistry ☐Removable Prosthodontics ☐Fixed Prosthodontics
☐Periodontics ☐Endodontics ☐Orthodontics
☐Oral Surgery ☐Pediatric Dentistry ☐Pharmacotherapeutics
☐Biomaterial ☐Pain Control ☐Oral medicine
☐Biostatistics & Methods of Scientific Research
☐Professional Ethics & Law, Practice Management

14- **Choose from the list <u>the top 3 reasons</u> for why would you be late or absent from a lecture?**

☐The lecture is before or after an examination
☐Early morning class (8-9 am)
☐Late class (3-5 pm)
☐If I have two or more-hour breaks before or after a class
☐Lecture material is available through Blackboard, video or voice recording or another source
☐The lecturer is below expectations (e.g. read directly from the slides, lacks clarity and organization)
☐The lecturer is not strict about student attendance
☐I am not interested in a specific topic
☐I can learn the subject of the day better by spending the same time studying at home
☐I don't wake up early
☐Difficulty with transportation

☐I don’t feel attending classes benefits me

☐other, please explain. . . . .
